# Pertussis resurgence and epidemiology of fully vaccinated cases in eastern China: evidence for vaccination timing

**DOI:** 10.3389/fpubh.2025.1677699

**Published:** 2025-10-07

**Authors:** Kai Gao, Yao Zhu, Yang Zhou, Linling Ding, Hui Liang, Hanqing He, Xiaohua Qi

**Affiliations:** ^1^Department of Immunization Program, Zhejiang Provincial Center for Disease Control and Prevention, Hangzhou, China; ^2^Zhejiang Key Lab of Vaccine, Infectious Disease Prevention and Control, Hangzhou, China

**Keywords:** pertussis, resurgence, epidemiology, vaccination, children, China

## Abstract

**Objectives:**

This study aimed to comprehensively analyze the epidemiology of pertussis based on case characteristics and vaccination history in Zhejiang Province.

**Methods:**

We analyzed clinically diagnosed and laboratory-confirmed pertussis cases (aged 0–18 years old) reported in Zhejiang Province from January 2016 to December 2024. Vaccination history data were matched from the Zhejiang Provincial Immunization Information System.

**Results:**

A total of 63,664 pertussis cases were identified in Zhejiang Province, China. The annual incidence of pertussis among individuals aged 0–18 years increased gradually from 2016 (1.46/100,000) to 2023 (14.22/100,000), followed by a drastic surge to 512.56/100,000 in 2024. During the resurgence, incidence rose most substantially in kindergarten (3–6 years) and lower elementary (7–9 years) children, surpassing the incidence of infants in 2024. Among 55,968 identified pertussis cases with data on vaccination status, the proportion of fully vaccinated cases increased from 12.50% in 2016 to 79.24% in 2024. Among 43,004 fully vaccinated cases, kindergarten children accounted for the highest proportion (51.27%), followed by the lower elementary children (34.58%). Regarding infection timing, 68.4% of fully vaccinated cases occurred after 6 years old, whereas 31.6% occurred before 6. The median interval between last vaccine dose and disease onset was 61.45 months (interquartile range: 48.15–79.47 months).

**Conclusion:**

Our findings underscore the critical need for booster vaccination at 4–6 years, and highlight the necessity for future research to focus on vaccine safety, effectiveness, and cost-effectiveness in optimizing vaccination strategies.

## Introduction

Pertussis (whooping cough) is a highly contagious respiratory disease primarily caused by *Bordetella pertussis*, and characterized by severe paroxysms of repeated coughs that can last for weeks or longer (1). Historically, the introduction of whole-cell pertussis vaccines (wP) in the mid-20th century led to a marked reduction in pertussis incidence and mortality (2). However, since the 1980s, multiple countries with high vaccine coverage have experienced a resurgence of pertussis cases (3). This epidemiologic shift is attributed to several factors including genetic mutation of *Bordetella pertussis*, suboptimal vaccine-induced immunity, enhanced surveillance sensitivity, and improved diagnostic capabilities (4). Since 2023, a global resurgence of pertussis has occurred, evidenced by large-scale outbreaks across China and other nations, posing a significant public health challenge (5–8).

Vaccination is the most effective preventive measure for pertussis. Before 2025, China’s immunization strategy included four doses of diphtheria, tetanus, and acellular pertussis (DTaP) vaccine at 3, 4, 5, and 18 months. Despite high vaccination coverage, epidemiological data have revealed rising disease burden among school-aged children, who usually had completed this primary series (2, 9). In response, China implemented a revised immunization strategy in 2025, adding a booster DTaP dose at age six, aiming to protect school-aged children (10). However, previous seroepidemiological study indicating the pertussis-specific antibody induced by four dose DTaP wane before 6 years old (11–13). Should the booster vaccination be administered younger than 6 years old, just like other countries have done? (14–16) The distribution of vaccination history among pertussis cases is important to answer this question. However, this evidence was limited in China (17). Furthermore, under the previous national vaccination strategy, detailed epidemiological data on fully vaccinated cases (those infected after completing the 4-dose series) remain scarce.

This study aims to analyze pertussis epidemiology in Zhejiang Province from 2016 to 2024, focusing on temporal trends of incidence, distribution of vaccination history, and epidemiological characteristics of fully vaccinated cases. Our findings will advance understanding of pertussis epidemiology and help optimize future vaccination strategies to mitigate pertussis burden.

## Methods

### Data sources

Pertussis case data were obtained from the National Notifiable Disease Surveillance System (NNDSS). In China, pertussis is classified as a Category B notifiable infectious disease, requiring mandatory reporting by all healthcare institutions through the NNDSS. For this study, we selected pertussis cases reported in NNDSS with residence addresses in Zhejiang province and onset dates between January 1, 2016 and December 30, 2024. Age-, year-, and sex- specific population data were also extracted from the NNDSS.

Vaccination history data were retrieved from the Zhejiang Provincial Immunization Information System (ZPIIS). Since its full implementation in 2016, ZPIIS has achieved province-wide coverage across all vaccination service providers, systematically documenting complete demographic profiles and vaccination records for all recipients. Case vaccination history was obtained through record linkage using national ID numbers, patient names, and parental names as matching criteria.

### Case definition

This study included only clinically diagnosed and laboratory-confirmed pertussis cases. The diagnostic criteria underwent minor modifications during the study period. From January 2016 to November 2023, the diagnostic criteria for pertussis cases across all healthcare institutions followed the Diagnostic Criteria for Pertussis (WS 274–2007) (18), as follows: ① clinical diagnosed cases: patients presenting with paroxysmal or spasmodic cough persisting for ≥2 weeks, or (for infants) cough accompanied by recurrent apnea, cyanosis, bradycardia, or choking episodes, along with markedly elevated peripheral white blood cell count with lymphocytosis; ② laboratory-confirmed cases: isolation of *Bordetella pertussis* from sputum or nasopharyngeal secretions, or a ≥ 4-fold increase in pertussis-specific antibody titers between acute and convalescent serum samples. Since December 2023, diagnosis adhered to the Guidelines for Pertussis Diagnosis and Treatment (2023 Edition) (19), which incorporated positive nucleic acid testing for *Bordetella pertussis* as an additional laboratory confirmation criterion. We included cases aged ≤18 years only in this study, due to insufficient cases reported in individuals aged ≥19 years before 2024. Fully vaccinated cases were defined as those occurring 14 days after the completion of the 4-dose DTaP vaccination series.

### Statistical analysis

Annual and age-specific incidence rates (per 100,000 person-years) were calculated using case counts and corresponding demographic data. For trend analysis, a joinpoint regression model was used (20, 21), which managed to fit a series of joined line segments to the trends of incidence rates. A logarithmic transformation of the rates and the default parameters were selected as model options. The average annual percent change (AAPC) and its corresponding 95 95% confidence intervals (95% CI) were calculated to estimate the direction and magnitude of trends. Due to a substantial increase observed in 2024, AAPC was calculated separately for 2016–2023 and 2016–2024.

The proportion of vaccination status was calculated based on cases with available immunization records in ZPIIS. Distributions of fully vaccinated cases were summarized by year, sex, and age group, with frequencies and percentages reported. Chi-square test was employed to assess differences in the sex and age distribution of fully vaccinated cases across different years. Age distribution of fully vaccinated cases was presented as count, percentiles and visualized using probability density plots. For the fully vaccinated cases, the intervals between the fourth dose and disease onset were determined and visualized using boxplot.

Data analyses and visualization were performed using R software (version 4.4.1). The joinpoint regression models were performed using the joinpoint regression Program (version 5.2.0.0). All statistical tests were two sided, and *p* values <0.05 were regarded as statistically significant.

## Results

During the surveillance period from 2016 to 2024, a total of 63,664 pertussis cases were identified among individuals aged 0–18 years in Zhejiang Province, China. The annual incidence of pertussis among individuals aged 0–18 years increased gradually from 2016 (1.46/100,000) to 2023 (14.22/100,000), followed by a drastic surge to 512.56/100,000 in 2024. During 2016–2023, infants (<1 year) consistently had the highest incidence among all age groups. In contrast, during the 2024 epidemic surge, the incidence in infants (738.63/100,000) was exceeded by both kindergarten children (3–6 years: 975.78/100,000) and lower elementary children (7–9 years: 942.79/100,000) (Table 1). The AAPC for all cases was 38.20% (95% CI: 6.81, 151.76) during 2016–2023, and 77.50% (95% CI: 34.05, 233.69) during 2016–2024. Age-specific trends during 2016–2023 showed significant increases in kindergarten (AAPC: 56.63, 95% CI: 25.38, 192.69), lower elementary (AAPC: 76.60, 95% CI: 22.73, 363.73), and upper elementary children (AAPC: 76.73, 95% CI: 18.64, 453.96). From 2016 to 2024, pertussis incidence increased significantly across all age groups, with AAPC exceeding 200% in individuals ≥3 years old (Table 1).

**Table 1:** Incidence and trends by age group in Zhejiang Province.

Incidence and trends	Infant (<1 year)	Pre-Kindergarten (1–2 year)	Kindergarten (3–6 year)	Lower elementary (7–9 year)	Upper elementary (10–12 year)^*^	Middle school (13–18 year)^*^
Incidence, (1/100,000)						
2016	18.42	2.09	0.78	0.15	0.00	0.00
2017	41.66	3.37	1.06	0.10	0.16	0.00
2018	83.89	13.31	5.75	0.95	0.39	0.06
2019	64.00	11.25	7.23	1.80	0.62	0.11
2020	5.50	0.99	0.46	0.14	0.14	0.00
2021	16.39	1.56	5.99	7.67	1.78	0.06
2022	135.29	16.86	55.83	56.74	17.99	1.38
2023	71.95	10.22	24.80	21.65	4.99	0.45
2024	738.63	310.29	975.78	942.79	304.61	61.90
Trends, % (95% CI)						
AAPC, 2016–2023	14.22 (−17.07, 63.22)	11.06 (−25.15, 78.77)	**56.63 (25.38, 192.69)**	**76.60 (22.72, 363.73)**	**76.73 (18.64, 453.96)**	58.76 (−14.16, 686.21)
AAPC, 2016–2024	**35.73 (10.35, 117.80)**	**59.41 (17.13, 340.52)**	**247.44 (199.02, 757.99)**	**350.95 (264.67, 992.92)**	**348.77 (264.35, 1079.89)**	**481.03 (380.14, 2594.44)**

^*^ zero was not included in the joinpoint regression model.

AAPC, average annual percent change. Bold indicates that AAPC was statistically significant.

**Figure 1:**
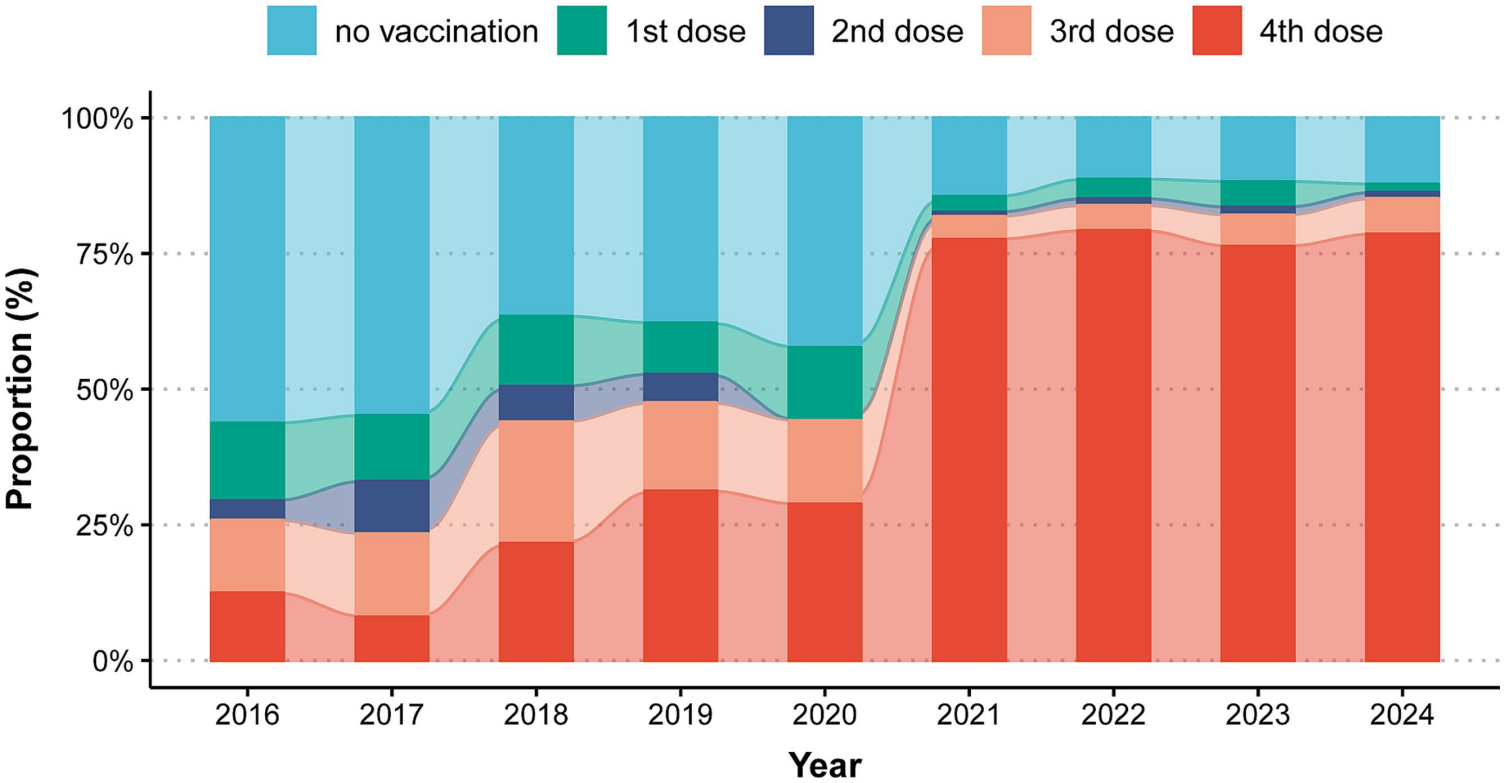
Proportion of vaccination status among pertussis cases in Zhejiang province (*n* = 55,968).

Among all reported pertussis cases, information on vaccination status was documented for 55,968 cases (87.91% of total cases). During 2016–2017, unvaccinated individuals constituted most (56.25%–54.84) reported cases, followed by individuals received 3 doses (13.39%–15.32), 1 dose (14.29%–12.10) and 4 doses DTaP (12.50–8.06%). From 2017 to 2020, the proportion of fully vaccinated cases (4 doses) increased gradually from 8.06 to 28.85%, whereas unvaccinated cases decreased from 54.84 to 42.31%. During 2021–2024, compared with 2020, fully vaccinated cases increased sharply and stabilized at 77.63–79.24%, with concurrent declines in other vaccination categories (Figure 1).

**Figure 2:**
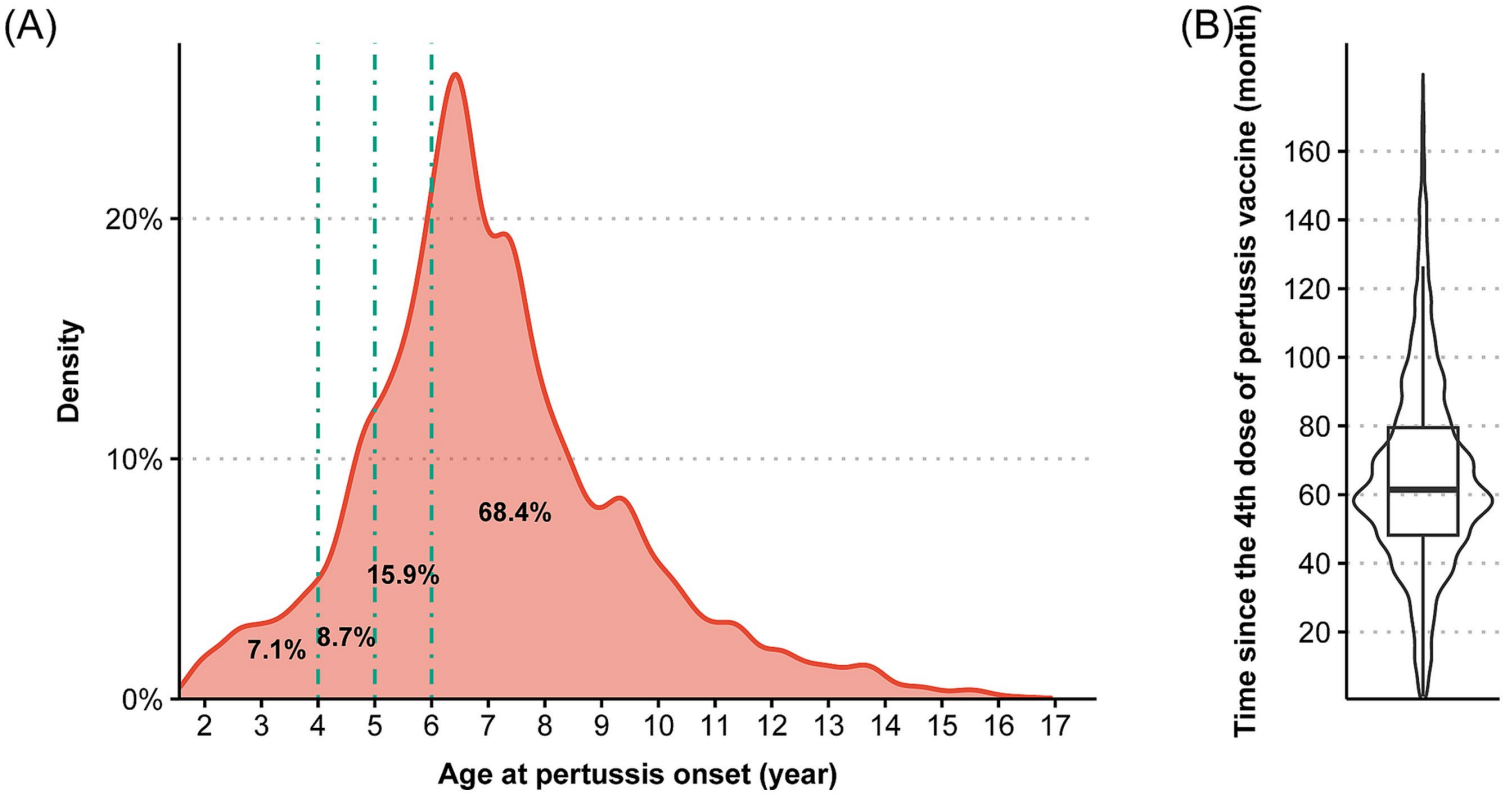
The distribution of **(A)** age at pertussis onset and **(B)** time between pertussis onset and the fourth dose of pertussis vaccine among pertussis cases finishing four dose vaccination (*n* = 43,004). ^*^Percentages in the probability density plot denote the proportion of cases falling between adjacent age cutoff lines.

Among the 43,004 fully vaccinated pertussis cases, Table 2 presents their age and sex distribution. During 2016–2024, 22,128 male cases (51.46%) and 20,876 female cases (48.54%) were reported, with a statistically significant temporal variation in sex distribution (*p* = 0.01). Females represented a slightly higher proportion of cases before 2021, whereas males showed a marginally greater proportion after 2022. Age distribution analysis showed that the kindergarten children constituted the highest proportion of fully vaccinated cases (51.27%). From 2016 to 2024, this group consistently accounted for over half of all fully vaccinated cases. Pre-kindergarten children (1–2 years) was the second largest proportion during 2016–2020. However, cases among lower elementary children increased substantially, becoming the second largest group during 2021–2024. Density plot showed that 68.4% of fully vaccinated cases occurred after 6 years old, 15.9% occurred at 5 years old, 8.7% occurred at 4 years old, and 7.1% occurred between the fourth dose vaccine and 4 years old (Figure 2A). The median interval between last vaccine dose and disease onset was 61.45 months (interquartile range: 48.15–79.47 months) (Figure 2B).

**Table 2:** Annual occurrence of fully vaccinated pertussis cases in Zhejiang province.

Characteristics	Total (*n* = 43,004)	2016 (*n* = 14)	2017 (*n* = 19)	2018 (*n* = 137)	2019 (*n* = 201)	2020 (*n* = 15)	2021 (*n* = 295)	2022 (*n* = 2,861)	2023 (*n* = 1,164)	2024 (*n* = 38,298)
Sex, *n* (%)										
Male	22,128 (51.46)	4 (28.57)	9 (47.37)	66 (48.18)	95 (47.26)	6 (40.00)	143 (48.47)	1,441 (50.37)	597 (51.29)	19,767 (51.61)
Female	20,876 (48.54)	10 (71.43)	10 (52.63)	71 (51.82)	106 (52.74)	9 (60.00)	152 (51.53)	1,420 (49.63)	567 (48.71)	18,531 (48.39)
Age, year, *n* (%)										
Pre-Kindergarten (1–2 year)	1,374 (3.20)	5 (35.71)	6 (31.58)	36 (26.28)	48 (23.88)	5 (33.33)	10 (3.39)	84 (2.94)	39 (3.35)	1,141 (2.98)
Kindergarten (3–6 year)	22,049 (51.27)	7 (50.00)	12 (63.16)	82 (59.85)	121 (60.20)	8 (53.33)	130 (44.07)	1,454 (50.82)	652 (56.01)	19,583 (51.13)
Lower elementary (7–9 year)	14,869 (34.58)	2 (14.29)	1 (5.26)	15 (10.95)	23 (11.44)	1 (6.67)	126 (42.71)	1,014 (35.44)	380 (32.65)	13,307 (34.75)
Upper elementary (10–12 year)	3,728 (8.67)	0 (0.00)	0 (0.00)	3 (2.19)	7 (3.48)	1 (6.67)	27 (9.15)	273 (9.54)	80 (6.87)	3,337 (8.71)
Middle school (13–18 year)	984 (2.29)	0 (0.00)	0 (0.00)	1 (0.73)	2 (1.00)	0 (0.00)	2 (0.68)	36 (1.26)	13 (1.12)	930 (2.43)

## Discussion

This study comprehensively characterizes the epidemiology of pertussis in the Zhejiang Province, China, from 2016 to 2024, revealing a concerning disease resurgence and increasing burden among fully vaccinated children. Pertussis incidence increased substantially throughout the study period, particularly among children aged 3–12 years. The proportion of fully vaccinated cases demonstrated a marked increase and stabilized at >75% in recent years. Notably, among fully vaccinated cases, 68.4% occurred ≥6 years of age, and 31.6% occurred before age 6 years. These findings indicated a transformation of pertussis epidemiology in China, that may require reevaluation of existing prevention and control measures.

In Zhejiang province, the pertussis incidence increased greatly during 2016–2023 (from 1.46/100,000 to 14.22/100,000), and experienced a drastic increase in 2024 (512.56/100,000). This rise in pertussis infection has been replicated across many countries: France and the United States also experienced a resurgence of pertussis in 2024 (15, 22); China’s national surveillance echoes this pattern, showing a 23-fold case increase in early 2024 and unprecedented infant mortality (2, 9). Several interconnected factors may drive this resurgence, including genetic adaptations in *Bordetella pertussis* (23, 24), the replacement of whole-cell pertussis (wP) with shorter-protective acellular pertussis (aP) (11, 25), and the enhancements of detection and surveillance in China (2). Similar to global trends, a significant pertussis resurgence emerged post-COVID-19 pandemic in Zhejiang Province. This trend was likely driven by the relaxation of non-pharmaceutical interventions after the COVID-19 pandemic combined with immunity debt from reduced pathogen exposure (15, 22, 26–28).

Along with the resurgence, our results showed that the incidence rates among kindergarten-aged children (975.78/100,000) and lower elementary school children (942.79/100,000) increased drastically and reached their peak in 2024, surpassing rates among infants (738.63 per 100,000), demonstrating that school-aged children and adolescents now bear a higher pertussis burden of pertussis than infants. This epidemiological shift aligns with national disease burden patterns (29, 30) and mirrors the U.S. resurgence pattern observed during the 2000s (31), likely resulting from both genuine epidemiological transitions and enhanced surveillance capabilities (32). Specifically, school environments function as transmission amplifiers due to high-density student contact during classroom activities (33), and waning immunity from aP vaccines leaves older children vulnerable. Previous seroepidemiological studies demonstrated that anti-PT IgG seropositivity declined to 9.81% among 5–6-years-old children (11, 34). This rapid antibody decline, coupled with deficient Th1-dominated cellular immunity, compromises long-term protection and contributes to high incidence rates in this age group (35). Concurrently, improvements in diagnostics, surveillance systems, and clinician awareness have increased case detection, particularly among school-aged children historically underdiagnosed due to milder symptom presentation (36).

In current study, fully vaccinated cases increased from 12.50% of total cases in 2016 to 79.24% in 2024. Considering the high coverage of pertussis vaccine among children in China (>99%) (37), this result provided evidence that waning immunity may be an important driver of disease resurgence. This phenomenon mirrors experiences in multiple countries with high coverage of acellular pertussis (aP) vaccines (38). In China, evidence regarding the epidemiological distribution of fully vaccinated cases was lacking. We found the kindergarten children accounted for the highest proportion of fully vaccinated cases (51.27%), with the lower elementary children ranking the second (34.58%). This pattern also closely corresponds to the expected window of declining vaccine protection following the fourth dose, as most of fully vaccinated cases occurred 4–6 years after vaccination, corresponding to the age of kindergarten and lower elementary children (Figure 2B). These findings indicate that a booster dose following the primary 4-dose series is needed to provide adequate protection for school children.

Globally, nations have implemented strategic booster programs to reduce disease burden on children and adolescents: Germany administers boosters at 5–6 years to maintain protection before school entry (14); the United States and Canada recommend preschool boosters at 4–6 years following the infant series, alongside adolescent, adult, and maternal Tdap vaccination (16, 39); China’s revised immunization schedule effective at January 2025 (2, 4, 6, 18 months, and 6 years old) represents a significant step toward enhancing protection for young children and school-aged populations (10). However, our findings showed that 31.6% of breakthrough infections in fully vaccinated children occurred before age 6, highlighting a critical susceptibility gap during the kindergarten years when immunity is expected to have waned but the scheduled booster has not yet been administered. This suggests that while the new 6-year booster is a positive advancement, it may not adequately protect children during the peak risk period just prior to school entry. Unfortunately, there is no evidence on vaccine safety, immunogenicity, and effectiveness among Chinese children aged 4–5 years. Future research should address this gap to generate robust epidemiological evidence, thereby supporting policymakers in refining immunization strategies and reducing the growing pertussis burden.

This study has several strengths. Comprehensive surveillance data from NNDSS and ZPIIS systems enabled precise incidence calculations and vaccination history evaluation. For the first time, we present data on the distribution of vaccination history among fully vaccinated cases in Zhejiang Province, providing an innovative perspective for refining immunization strategies. However, several limitations merit consideration. First, despite rigorous matching algorithms, 12.1% of cases had missing immunization records, with higher rates observed among cases aged ≥13 years, which may lead to underestimation of the pertussis burden in this age group. Because this potential bias would likely result in a conservative estimate of waning immunity, our finding of increased susceptibility in older individuals further underscores the importance of booster immunization. Second, although pertussis resurgence occurred among adults in 2024, our analysis was constrained to cases aged ≤18 years due to insufficient cases reported before this year. Third, our analysis relied on reported pertussis cases. The increased surveillance sensitivity, especially the adoption of PCR testing in diagnosis, may have led to overestimating the true epidemiological resurgence. Moreover, the lack of population-based data precluded the assessment of vaccine effectiveness by dose completeness. Finally, caution is warranted when extrapolating Zhejiang-based findings to other Chinese provinces, given marked interprovincial heterogeneity in socioeconomic development and sociocultural contexts.

This study demonstrates pertussis resurgence in Zhejiang Province through epidemiological and vaccination history evidence. The rising incidence among kindergarten and elementary school children, coupled with the age distribution of fully vaccinated cases, highlights the importance of booster immunization for children aged 4–6 years old. Future evidence on vaccine safety, effectiveness, and cost-effectiveness in China is required to refine vaccination strategy and alleviate the growing pertussis burden.

## Data Availability

The datasets presented in this article are not readily available because of local policy reasons. Requests to access the datasets should be directed to Xiaohua Qi, xhqi@cdc.zj.cn.
